# Chicago Society of Opthalmology and Otology

**Published:** 1884-10

**Authors:** 


### SOCIETY PROCEEDINGS.

CHICAGO SOCIETY OF OPHTHALMOLOGY AND OTOLOGY.

*Illinois Charitable Eye and Ear Infirmary. April 8, 1884.*

Dr. Boyer was unanimously elected a member of the society. Dr. Holmes then referred to a case in which he had recently removed a splinter of steel from the vitreous by means of a Gluening's magnet. The incision in the sclerotic, through which the magnet was introduced, was made behind the ciliary region in the temporal portion of the globe, although the foreign body could be seen lying in the vitreous near the equatorial region of the opposite hemisphere. The lens was so far cataractous that the metal could not be precisely located either by oblique illumination or by the ophthalmoscope. Dr. Holmes believed the end of the magnetized probe could in this way be brought near the steel with less violence to the edges of the wound of the sclerotic, choroid, and uteria than if the wound had been made in the nasal portion of the globe. In a case in which, the lens being intact, the metal could be exactly located, it would be desirable to introduce the magnet as near the foreign body as possible, although the reporter thought there is liable to be more hemorrhage from the conjunction at the inner than at the external angle. The doctor thought that a crucial incision through the sclerotic, although advised, is objectionable, and called for the opinion of the society as to the relative advantage of the various kinds of incision.

Dr. E. J. Gardner thought a crucial incision would expose the eye to greater danger than a long linear incision, in the latter case there being but two instead of four flaps.

Dr. Montgomery expressed a similar opinion.

Dr. F. C. Hotz also advocated the linear incision, and in this connection reported the following case:

*Piece of Steel in Vitreous.*—August 29, 1882, I removed a piece of steel from the vitreous chamber with Gluening's magnet under conditions which proved a very good result as to the preservation of the sight. The steel entered through the sclerotic on the previous evening. Except the minute cut on the sclerotic, six mill. from the nasal border of the cornea, there was nothing abnormal with the eye. With the ophthalmoscope a lanceolated body was discovered suspended in the vitreous just above and behind the center; it presented a bright, shining, silvery surface, and looked very much in shape as a diminutive silver fish, the head turned downwards. A few dark streaks extended from the body to the sclerotic wound, otherwise the vitreous was clear; the fundus showed no lesion. The sight, I am sorry to say, was not tested. Under chloroform, the scleral wound was enlarged horizontally to two and one-half mill.; the edges were held apart with fine hooks, Gluening's magnet introduced, and, on the first attempt, the steel was brought out. It was six mill. long, one and one-half mill. broad, tapering off to sharp points, and weighed twenty milligrams. The wound was closed up by a conjunctival suture, and the patient put to bed with both eyes bandaged. He was kept abed in a dark room a whole week; no unfavorable reaction followed, but he could only count fingers at six feet. With the ophthalmoscope I discovered no disturbance in the vitreous, except two dark streaks from the wound to the center and an opaque transverse streak in the fundus above the papilla. During the second week the lower portion of the vitreous became very cloudy, and the opaque stripe in the fundus developed into a distinct retinal detached fold. Later, the obscuration of the vitreous consolidated into several separate clouds, between which portions of the fundus could be seen; some parts of it appeared normal, in some the retina was distinctly detached, and in some it seemed that the retina was separated from the choroid by a thin layer of serum. The patient was under observation three months. No material changes took place, and the sight did not improve.

Dr. G. T. Gardner then read a paper, entitled—

*Detachment of the Retina.*—After reporting a case of extensive detachment of the retina, in which total reapplication of the detached membrane took place, remaining *in situ* for two weeks, and then, without any perceptible cause, becoming detached again, the lecturer said:

"The literature on the etiology of detachment of the retina is so extensive that the scope of this paper will not permit me to mention all that has been written on the subject, but I will endeavor to record—not with the extension that they deserve—the theories which now seem to be most in favor:

"For the sake of clearness, these theories may be divided into three groups.

"The authors whose theories are comprised in the first group claim that it is a fluid secreted by the choroid, which, accumulating under the retina, produces the detachment.

"Those comprised in the second, that a lack of elasticity in the retina, as compared with the choroid and sclerotic, is the immediate cause.

"In the third group are those who look on a shrinkage of the vitreous as the direct cause of detachment.

"Among the latest articles on this subject, I will mention that of Professor Rava, who thinks that in a partial elongation of the choroid a distension of its elastic layer, and also of its hexagonal epithelium, takes place. The sclerotic also becomes thinned at this portion of the globe. This thinning of the membranes favors, according to him, the filtration of the lymph contained in Schevalbe's spaces, and facilitates its accumulation between the choroid and the retina.

"Brucherm, in a recent communication made to the Congress of Rouen, introduces, in the pathogenesis of the retina, the latest theories advanced by the physiologists about the secretion of the intravecular liquids. He claims that the choroidal epithelium is the secretory element. When this secretion is excessive it accumulates behind the retina, and detachment is produced. As causes of this hypersecretion, he mentions affections of the kidneys, rheumatism, etc.

"Galezowski, in an exhaustive article published in the *Receuil Ophthalmologie*, claims that a circumscribed choroido-cyclitis is the cause of detachment, but that a previous liquefaction of the vitreous must necessarily exist before a detachment takes place. To prove his theory (advanced first in 1873), he reports several interesting cases, and then appends a very instructive synoptic table of 649 cases. Of these 598 happened in myopic eyes, 51 in cases of hypermetropia and emmetropia. In 131 cases only could a rent be detected in the retina. In 120 he observed floating opacities in the vitreous. This author thinks that the presence of floating opacities in the vitreous is a sign of perforation or tearing of the retina.

"Of those who look on the pathological changes of the vitreous humor as the direct cause of detachment of the retina, Henri Muller should be first mentioned. In 1866, he made the assertion that in certain cases detachment of the retina was caused by shrinkage of the vitreous. Previous to this date, however, he had called attention to the fact of the vitreous possessing the property of diminishing in volume, through a process of shrinkage, which could end in a detachment of this humor from the retina. The first histological investigations in this phenomena were made by Irvanoff.

"Professor Leber read before the Ophthalmological Congress, at Heidelberg (1882), a very instructive paper, in which he explains the detachment of the retina by the so-called perforation theory. By this theory, the frequent phenomenon of sudden detachment of the retina, without a perceptible change in the tension of the eye-ball, is explained. The primary cause of the detachment is a localized alteration of the vitreous in contact with the retina, consisting in hypertrophy of the radial fibers, formation of fine bundles of connective tissue, which, in the process of contraction, exert a traction upon the retina, which finally produces a solution of continuity in this membrane, thus allowing the degenerated and liquified vitreous to pass between it and the choroid. In this process there is simply a change of locality of the fluid from the inner side to the outer; consequently, there is no cause for an increase of tension.

"Leber bases his theory upon the evidence brought to light by the examination of twenty-seven cases of detachment seen by him during the last two years. Of these, fifteen were 'fresh' cases, eleven from a few days to two weeks' duration. In eleven of these a perforation was demonstrated; in one it was suspected, while in the remaining three its existence could not be proved—neither excluded. In long-standing cases, perforations were not seen as often. In twelve cases, existing from ten months to ten years, a perforation was found only in three cases; in four, its existence seemed very probable; not found in five cases. Summing up the twenty-seven cases, a positive result was reached in fourteen; doubtful, four; negative, eight.

"We have now reviewed, though in a very succinct manner, the theories advanced to explain the causes of a disease with which we are unfortunately too familiar. But wherein lies the truth, is hard to tell. All the theories are made so plausible, by their propounders, that one is tempted to accept them as the truth, and turn away from that uncomfortable state of mind which to uncertainty is inherent. But, unexpectedly, some clinical fact jumps into the field, and overturns the whole theoretical structure, leaving us in the same perplexing situation from which we started. The truth is, although it may pain us to express it, that the etiology of detachment of the retina in myopic eyes, still remains shrouded in mystery.

"In the selection of a theory, we should be guided entirely by clinical experience, for I believe that a clinical fact is worth more than a hundred theories. Experimentation is worth but little in this line of investigation, because the process to which an eye has to be submitted, in order to bring about the condition which we find present in highly myopic eyes, is such that it renders the results untrustworthy. We know that foreign bodies thrust into the eye, experimentally or accidentally, will, through a process of inflammation and subsequent shrinkage of the vitreous detach the retina, but this fact can not explain the causes of detachment in myopic eyes, where absolutely no traumatism has existed. I performed a number of experiments on pigs' eyes, but here also the results were unreliable, not only on account of the post mortem changes which had taken place, but also because the condition of the vitreous is entirely different from that found in highly myopic eyes, and in attempting to render it liquid, or withdraw a portion of it, or inject (or rather attempt to inject) fluid under the retina, so much traumatism is necessary that reliable results are nearly impossible. Experiments may and do cast a little light upon the matter, but it is not sufficient to enable us to draw any positive deductions therefrom. Relying entirely on the clinical test, let us, by its light, examine these theories.

"The theory of subretinal effusion can explain certain varieties of detachments of the retina, if a pre-existent diminution in the volume of the vitreous is admitted. In a *gradual detachment* this theory explains matters in a satisfactory manner; but in a sudden detachment it does not. I cannot conceive how a vitreous, partially liquefied or not, should, as if by magic, decrease sufficiently in volume to allow another fluid to exude into a chamber limited by a resistant lining, and so do this *withont increasing at any time the tension.* This is contrary to all physical law, and, when we remember that the tension, far from being increased, is *diminished*, the conclusion forces itself upon our minds that we must look elsewhere for a more satisfactory explanation.

"We do not find it, I think, in Von Graefe's theory, in which the trouble is ascribed to a lack of elasticity of the retina as compared to that of the choroid and of the sclerotic. This theory does not stand the clinical test, for how can we admit this lack of elasticity in a membrane which gives such strong evidences of the contrary in its inflammatory conditions?

"Let us now turn to the theory based upon the shrinkage of the vitreous as a cause of detachment of the retina.

"Before entering fully into the matter, I will endeavor to explain the reason why the posterior portion of the vitreous, in highly myopic eyes, where sclerectasia posterior exists, is found to be in a fluid, degenerated condition, and also why, in such eyes, there is a tendency to shrinkage of the vitreous.

"The experiments performed by Max Knies, show that the vitreous receives its nutrition from the choroid; the liquid lymph passing through the retina, the external layers of which it also nourishes. At the posterior pole, according to this author, the nutrition is very slight, owing to the greater thickness of the retina at this place. As the retina becomes thinner, the amount of fluid which passes into the vitreous is larger, and it reaches its maximum at the most anterior portion of the choroid. Now, if in the physiological condition the nutrition of the posterior pole is very meager, it is but reasonable to deduct, that, if the choroid at this point is degenerated, the nutrition will decrease in proportion to such degeneration, reaching at last a point where the secretion of lymph is so poor that it lacks the power to constitute healthy vitreous humor. In this manner the liquefaction of the vitreous at the posterior pole of highly myopic eyes—mentioned by Alt—is easily explained. But even if the theory of Max Knies is not accepted—and we admit that the vitreous receives its nutrition only from the anterior portion of the choroid, the ciliary body—then, too, if there is a lack of nutrition, the first part to suffer will be that further removed from the source of nutrition; in other words, that portion of the vitreous nearest the posterior pole. It is a fact that highly myopic eyes, in which degeneration of the choroid or sclerectasia posterior is present, are not healthy, and the liquefaction of the vitreous is a proof in itself of the poor nutrition that it has received, be its source from the ciliary body or from the whole choroid. The natural consequence of poor nutrition is shrinkage, diminution in volume, atrophy, etc. These premises once accepted, we find that Leber's theory will explain, in a satisfactory manner, the phenomena which take place in detachment of the retina—a shrinking vitreous, in the anterior portion of which a radial formation exists. (Leber.) The adhesions formed by a diseased vitreous, at those points where it is still in contact with the retina, a liquefaction of the vitreous beginning at the posterior pole, and gradually advancing toward the anterior portion of the eye, are the immediate causes of the catastrophe, which only needs one step more to take place. The traction on the retina increases as the shrinkage progresses, thus giving rise to the metamorphopsia and other prodromic symptoms. The continued and even increasing traction, or a sudden jar, overcomes the resistance of the already strained retina; it tears the lympid vitreous, enters behind the membrane, and the detachment is effected.

"Dr. Galezowski, in his excellent article, above referred to, states that he only found a rent in the retina in 131 cases, and that floating opacities were seen in but 120; he therefore claims that the presence of floating opacities, in a case of detachment of the retina, points to the existence of a rent in this membrane, and *vice versa*, that the absence of these opacities is a sign that no tearing has taken place. This statement does not bear the clinical test, however, for we all have seen cases where floating opacities in the vitreous long precede detachment of the retina. The simple fact that the rent could not be seen, does not, in my opinion, prove that it did not exist, for it might well happen that it was so situated that it could not be seen; indeed, I believe that in many cases they escape because they are so located that the folds of the detached retina entirely hide it from view, and, furthermore, it is not at all necessary for the solution of continuity to be large. The retina tears in a manner very similar to thoroughly soaked blotting paper, as I have been able to convince myself, by tearing the fresh retina under the microscope. There is a moment before the regular separation of parts takes place when a liquid can easily penetrate through it, and as the moment that the retina becomes detached the traction from the vitreous ceases, the rent may remain stationary, and not acquire such proportions as to render it visible with the ophthalmoscope. I do not consider the case which the same author reports, in which a small opacity was seen engaged in a perforated retina, working its way into the vitreous, as a proof that the opacities are formed only in the subretinal fluid.

"There is another form of detachment of the retina, the etiology of which is made clear by the theory of shrinkage of the vitreous, viz.: detachment in folds. In this form, the subretinal effusion theory utterly fails to explain the process; for a liquid secreted under a membrane, which is equally adherent throughout, would naturally expand in a circular form, the detachment would therefore commence at the center of the figure, and gradually creep on to the periphery. On the other hand, nothing easier to account for than a fold being raised by a traction exerted from within. The process can be easily demonstrated, even in eyes where the vitreous is not degenerated, by introducing a very fine pair of forceps into the vitreous chamber, firmly closing them and gently withdrawing them. In this manner I have been able to produce beautiful specimens of detachment in folds.

"I am perfectly aware that this theory does not answer in every case, and that the clinical history of certain cases will militate against it; but I firmly believe that in those cases of detachment of the retina, happening in highly myopic eyes, the immediate cause is the traction exerted by a degenerated and shrinking vitreous; and no other theory, that I know of, can explain in a rational manner one of its most constant phenomena, viz.: the suddenness of the detachment. Neither can any of the other theories explain in a rational manner how there can exist a fluid behind and another in front of the retina without a perceptible increase of tension."

This paper called forth a long discussion on the merits of pilocarpine treatment. Almost every member had given this remedy a trial; all were unanimous as to its inefficacy in producing a permanent cure.

Dr. Montgomery exhibited a new self-registering perimeter, by M. Blitz (Upsala). It is fixed to the back of the chair in which the patient sits. The test object is carried by a bent arm coming from behind, and is suspended before the patient's eye.* B. B.

* For a more detailed description, the reader is referred to the original article in the *Centralblatt fur prakt. Augenheilk.*, August, 1882, p. 251.

